# Quality evaluation of image‐based iterative reconstruction for CT: Comparison with hybrid iterative reconstruction

**DOI:** 10.1002/acm2.12597

**Published:** 2019-05-02

**Authors:** Hiroki Kawashima, Katsuhiro Ichikawa, Kosuke Matsubara, Hiroji Nagata, Tadanori Takata, Satoshi Kobayashi

**Affiliations:** ^1^ Faculty of Health Sciences, Institute of Medical, Pharmaceutical and Health Sciences Kanazawa University Kanazawa Japan; ^2^ Section of Radilogical Technology, Department of Medical Technology Kanazawa Medical University Hospital Uchinada Kahoku Japan; ^3^ Department of Diagnostic Radiology Kanazawa University Hospital Kanazawa Japan

**Keywords:** computed tomography, image quality, noise reduction, iterative reconstruction, dose reduction

## Abstract

The purpose of this study is to evaluate the physical image quality of a commercially available image‐based iterative reconstruction (IIR) system for two object contrasts to resemble a soft tissue (60 HU) and an enhanced vessel (270 HU), and compare the results with those of filtered back projection (FBP) and iterative reconstruction (IR). A 192‐slice computed tomography (CT) scanner was used for data acquisitions. IIR images were processed from the FBP images. Task‐based in‐plane transfer function (TTF) and slice sensitivity profile (SSP_task_) were measured from rod objects inside of a 25‐cm diameter water phantom at four dose levels (2.5, 5, 10, and 20 mGy). Noise power spectrum (NPS) was measured from the water‐only part. System performance (SP) function was calculated as TTF^2^/NPS over FBP, IR, and IIR for comparison. In addition, an image subtraction was performed using images of rod objects, a bar‐pattern phantom, and a clinical abdomen case to observe the noise reduction performance of IIR. As a results, IIR mostly preserved TTF and SSP_task_ of FBP, whereas IR exhibited enhanced TTF at 10 and 20 mGy for 60 HU contrast and at all doses for 270 HU contrast. SP of IIR at 2.5, 5, 10 mGy (half doses) were similar to those of FBP at 5, 10, 20 mGy, respectively. IR exhibited enhanced SP at medium to high frequencies. The subtracted images showed weak remained edge signals in the bar‐pattern and abdominal images. In conclusion, IIR uniformly improved the task‐based image quality of FBP over the entire frequency range, whereas IR improved the characteristics over medium to high frequencies. The dose reduction potential of IIR estimated from SP is approximately 50%, when allowing the slight signal reductions.

## 1 INTRODUCTION

Computed tomography (CT) imaging is widely used in general clinical practice. However, the radiation dose required for CT can be considered high, and patients are usually concerned about consequences such as cancer incidence. To reduce the dose while preserving image quality, various noise reduction techniques have been proposed.^1^ For instance, CT vendors usually offer iterative reconstruction (IR) algorithms for CT dose reduction, noise mitigation, and delivering a suitable image quality for clinical use.^2^, ^3^, ^4^

Although IR allows dose reduction in the most recent CT systems, some systems are not endowed with such techniques, and the benefits of dose reduction cannot be obtained for patients who are examined using these systems. To overcome this limitation, an image‐based IR system (IIR) (SafeCT, Medic Vision Imaging Solutions Ltd., Tirat Carmel, Israel), has been recently developed for clinical use and offers a vendor‐neutral technique for processing filtered back projection (FBP) images from CT scanners.^5^, ^6^ This system performs a three‐dimensional nonlinear denoising of FBP images and has been evaluated on CT examinations by comparing full‐dose and processed half‐dose images to verify dose reduction^5^ and against existing IR techniques.^6^ Although its effectiveness for clinical use has been confirmed, image characteristics such as noise and in‐plane and longitudinal resolutions have not been evaluated. Determining these characteristics can help to quantitatively assess this image‐based system and estimate the dose reduction capability.

The quality of IR images is affected by nonlinear characteristics, and spatial resolution varies according to noise level (related to radiation dose level) and target object contrast.^7^, ^8^ Thus, it is difficult to adapt conventional measuring methods to this type of images, because these methods assume FBP images with linear characteristics. Consequently, task‐based methods that use specific object contrasts and radiation doses have been proposed to consistently evaluate the quality of images processed using IR.^2^, ^3^, ^7^, ^8^, ^9^ In this study, we evaluated the physical image quality of IIR using a phantom and including objects with two different contrasts scanned at four dose levels, and compared the results with those of a state‐of‐the‐art IR technique.

## 2 MATERIALS AND METHODS

### 2.1 CT system and IR

For acquiring CT images, we employed a SOMATOM Force dual source CT scanner (Siemens Healthcare, Erlangen, Germany) equipped with advanced modeled IR (ADMIRE), which has five noise reduction levels from 1 to 5, with 5 corresponding to the most intense noise reduction. The CT images considered for comparison were FBP, ADMIRE and IIR. IIR images were processed from the FBP images.

### 2.2 Measurement setup

**Figure 1:**
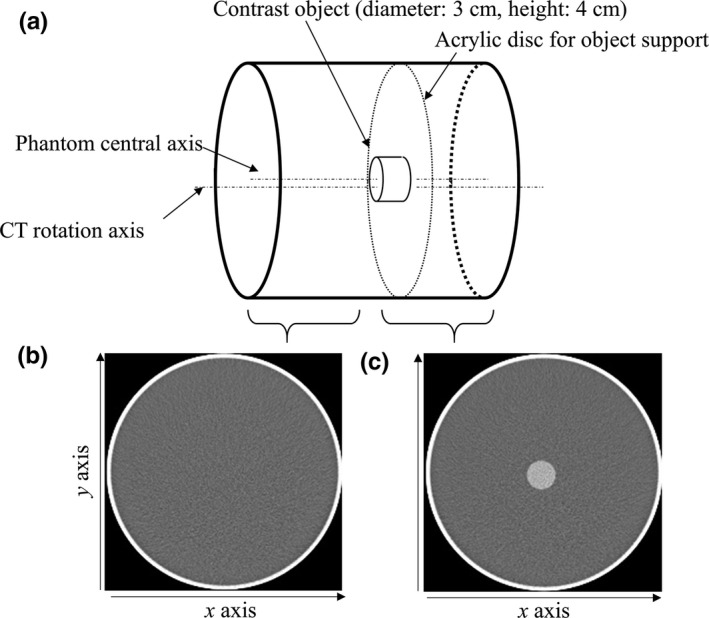
Experimental setup. (a) Phantom setup for measuring NPS and in‐plane TTF. Sample images to determine (b) NPS and (c) TTF

We used two rod‐shaped objects with 60 and 270 HU contrasts at 120 kV for measuring in‐plane task‐based transfer function (TTF), which has been employed for spatial resolution measurements of images processed with IR.^7^, ^8^ Each object with diameter of 3 cm and height of 4 cm was placed in a cylindrical acrylic case with diameter of 25 cm filled with water, as shown in Fig. 1(a). In this study, we approximated the rod size of the ACR phantom and used two contrasts of 60 and 270 HU to resemble a soft tissue and an enhanced vessel with a 12 mg iodine (mgI)/ml concentration, respectively. Moreover, the phantom diameter resembled the absorption of adult abdomen.^10^

The rod containing iodine for this study was commercially available as a custom order supplied by the phantom manufacturer Kyoto Kagaku Corporation (Kyoto, Japan). The central axis of the phantom was accurately positioned in parallel to the rotation axis of the CT system with a 10 mm offset in the y‐axis direction to avoid the specific modulation transfer function induced when the central axis matches the rotation axis of the CT system.^8^ The region of the phantom without the rod was used for measuring the noise power spectrum (NPS).

**Figure 2:**
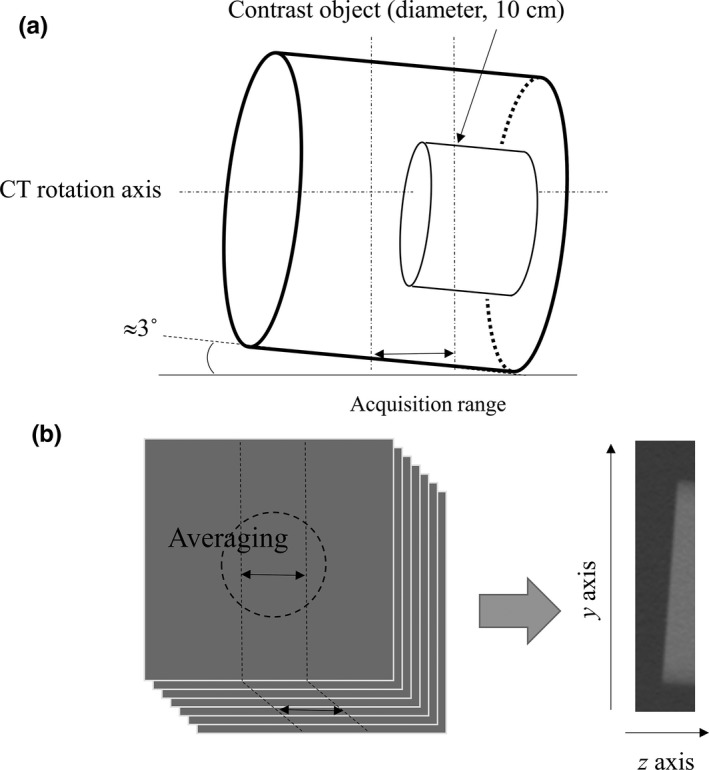
Experimental setup for imaging. (a) Phantom setup for measuring the task‐based slice sensitivity profile SSP_task_. (b) Image averaging of sagittal images from a stack of axial images of the phantom. SSP_task_ was measured from the slanted edge in the sagittal image using a conventional edge detection method

Two other rods with diameter of 10 cm and contrasts of 60 and 270 HU were used for measuring task‐based slice sensitivity profile (SSP_task_). The objects were placed in the phantom as shown in Fig. 2(a). The phantom was tilted by approximately 3° with respect to the rotation axis to apply an established edge method that provides a sufficiently fine effective sampling for Fourier analysis.^11^ An averaged sagittal image was created from a stack of axial images, as shown in Fig. 2(b), and SSP_task_ was measured from the object edge formed by the top surface of the rod.

### 2.3 Data acquisition

The imaging conditions were as follows: applied voltage of 120 kVp, rotation time of 0.5 s, pitch factor of 0.6 using a detector of 196 × 0.6 mm. The CT images obtained from FBP and ADMIRE were reconstructed with a display field of view of 250 mm, nominal slice thickness of 1 mm, and abdominal standard reconstruction kernel Br40d. The noise reduction level of ADMIRE was set to 3 and 5 (ADMIRE 3 and ADMIRE 5, respectively). The radiation doses in volume CT dose index CTDI_vol_ were set to 2.5, 5, 10, and 20 mGy for evaluating dose (noise) dependencies of image quality. A dedicated SafeCT workstation was used to process the FBP images. The abdomen filter option in SafeCT was set to default from the five noise reduction strengths, namely, Sharp+, Default, Soft+, Soft++, and Soft+++, with the latter being the strongest.

### 2.4 In‐plane TTF

TTF was determined from disc images of each rod. Image averaging can effectively improve the accuracy of transfer function measurements, and suitable contrast‐to‐noise ratios values should be above 25.^8^ Hence, we used between 150 and 500 images obtained from 5 to 15 acquisitions. Then, a one‐dimensional edge spread function from an averaged disc image was obtained using the circular edge method proposed by Richard et al.^7^ We set the bin width to one‐fifth of the pixel pitch to create equidistant data for the edge spread function and reduce noise.

### 2.5 Noise power spectrum

The NPS was determined from the central 256 × 256 pixels from the area of the phantom images that did not contain the rod and by using the radial frequency method based on the two‐dimensional Fourier transform. The two‐dimensional NPS measurements were radially averaged and split into 40 frequency bins. To reduce the NPS variability, the results from 80 consecutive images were averaged.^2^, ^12^

### 2.6 System performance function

Samei and Richard used the following detectability index, d', to assess the IR techniques' imaging performance:

(1) $$\left[{\text{d}}^{{}^{\prime }2}=\int \frac{\text{TT}{\text{F}}^{2}\left(\text{u}\right)}{\text{NPS}\left(\text{u}\right)}{\text{S}}^{2}\left(\text{u}\right)\text{du\textbackslash{};,}\right]$$

where u denotes the spatial frequency and S(u) is the spectrum of the signal to be detected. The d′^2^ value is a figure of merit that incorporates square of system performance (SP) TTF^2^(u)/NPS(u) and imaging task S^2^(u).^2^ This index is similar to the prewhitening signal‐to‐noise ratio that is based on an ideal observer model.^13^

In this study, we focused on this SP function expressed as

(2) $$\text{S}{\text{P}}^{2}\left(\text{u}\right)=\frac{\text{TT}{\text{F}}^{2}\left(\text{u}\right)}{\text{NPS}\left(\text{u}\right)}$$

Since the TTFs we measured were specific for rod objects with the soft tissue and iodine contrasts presenting circular edges, we used the SP function to evaluate the noise reduction performance for the specific conditions that did not cover various contrasts of tissues such as bones and fats in clinical CT images.

### 2.7 Slice sensitivity profile

From the longitudinal edge surface in the phantom shown in Fig. 2(a), we measured the longitudinal edge spread functions as follows. The averaged sagittal image was obtained by the process illustrated in Fig. 2(b) without considering pixel interpolation. In the region of interest containing the edge, a synthetic edge profile was created using the edge method established in previous studies.^11^ Both the averaging and edge synthesizing effectively reduce noise, and the tilting with respect to the z axis provides an oversampled profile to accurately detect the edge. The SSP_task_ was calculated from the derivative of the edge spread function, and then the full width at half maximum (FWHM) was determined from the obtained SSP_task_.

### 2.8 Image subtraction for evaluating noise reduction

We used a subtraction technique based on FBP‐processed images for IIR to determine noise reduction. The rod images for TTF at 10 mGy, a bar‐pattern phantom image, and clinical abdominal CT images were considered. The bar‐pattern phantom was placed horizontally and vertically in the cylindrical phantom instead of the rod objects, to observe in‐plane and longitudinal signal preservations, respectively. This phantom consisted of six segments with the bar widths of 0.5–5.0 mm, corresponding frequencies 0.1–1.0 cycles/mm. The phantom was scanned at 120 kV with CTDI_vol_ of 10 mGy. For the longitudinal direction, the coronal multi planer reconstruction images were reconstructed using FBP, IIR, and subtracted data. The clinical abdominal images were obtained using the SOMATOM Force CT system at 120 kV with CTDI_vol_of 25 mGy. The bar‐pattern and abdominal images were reconstructed with a nominal slice thickness of 1 mm, a display field of view of 250 mm, and reconstruction kernel Br40d. The use of the clinical images was approved by the institutional ethics committee (No. 766‐1). Subtraction was not adequate for ADMIRE images because it produced conspicuous patterns possibly by object edge alternations related with spatial resolution variations and object contrast differences by varying partial volumes.

## 3 RESULTS

### 3.1 In‐plane TTF

**Figure 3:**
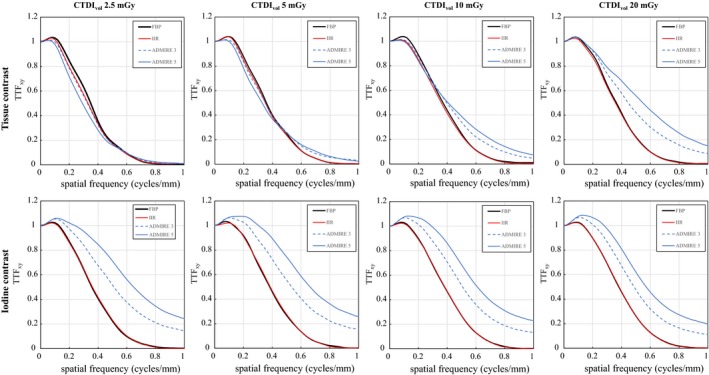
In‐plane TTF for doses of 2.5, 5, 10, and 20 mGy with tasks of soft‐tissue contrast and iodine contrast. ADMIRE 3 and ADMIRE 5 correspond to strength levels of 3 and 5 for ADMIRE.

Figure 3 shows the resulting in‐plane TTF. For the soft‐tissue contrast, IIR exhibits similar TTF as FBP at radiation doses of 5, 10, and 20 mGy, and slightly lower values at 2.5 mGy. ADMIRE exhibits a high dependency on the radiation dose, with a lower TTF_xy_ of ADMIRE 5 at 2.5 mGy and higher TTF_xy_ at 10 and 20 mGy compared to FBP. The resolution increase in ADMIRE 5 was stronger than that of ADMIRE 3. For iodine contrast, IIR almost completely preserved the TTF of FBP regardless of radiation dose. In contrast, ADMIRE exhibits significantly increased TTF at all the evaluated doses.

### 3.2 Noise power spectrum

**Figure 4:**
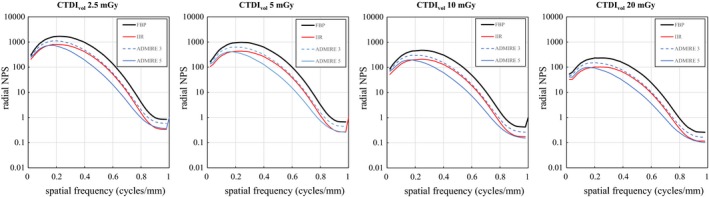
NPS for doses of 2.5, 5, 10, and 20 mGy. Each NPS curve of IIR is shifted downward and almost parallel to the corresponding curve of FBP, whereas ADMIRE decreases noise mainly at medium to high frequencies, and this trend is more notable with increasing strength.

Figure 4 shows the NPS for the evaluated techniques and conditions. Both IIR and ADMIRE achieved notable noise reduction compared to FBP. In addition, IIR shows almost constant NPS mitigation over the entire frequency range. When using ADMIRE, the NPS decreased with its increasing strength level, and the distribution was changed to low‐frequency dominant, especially for ADMIRE 5. The NPS peak frequencies, which can be used to evaluate noise texture change,^9^, ^14^, ^15^ were 0.24, 0.18, 0.16, and 0.24 cycles/mm for FBP, ADMIRE 3, ADMIRE 5, and IIR, respectively, at CTDI_vol_ of 10 mGy. Similar results were obtained at 5 and 20 mGy. However, at 2.5 mGy, the frequencies were 0.22 cycles/mm for FBP and 0.18 cycles/mm for ADMIRE 3, ADMIRE 5, and IIR, indicating a peak frequency shift for IIR only at this lowest dose.

### 3.3 System performance function

**Figure 5:**
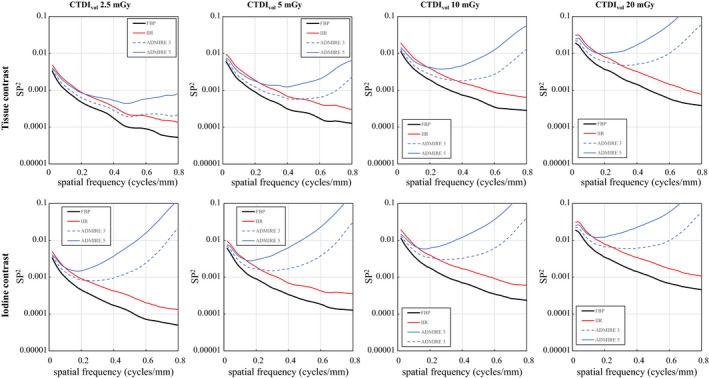
Results of system performance function SP(u) ^2^ calculated as TTF^2^/NPS for doses of 2.5, 5, 10, and 20 mGy with tasks of tissue contrast an iodine contrast.

Figure 5 depicts SP function at the four dose levels for the evaluated techniques and images. IIR clearly improved SP^2^ compared to FBP, as shown in the SP curve nearly parallel and above that of FBP at every dose level, indicating a uniform improvement of IIR over the entire frequency range. Specifically, SP^2^ of IIR at the lowest frequency of 0.02 cycles/mm, at which the smallest difference between IIR and FBP was obtained, increased by 47.5%, 56.6%, 68.6%, and 63.9% compared with FBP for doses of 2.5, 5, 10, and 20 mGy, respectively. Therefore, SP^2^ of IIR at 2.5, 5, and 10 mGy was almost equal to that of FBP at 5, 10, and 20 mGy, respectively, indicating a dose reduction capability of approximately 50% when using IIR.

On the other hand, SP of ADMIRE strongly depends on the dose level and its trend changed from decreasing to increasing at a certain frequency that varied with both the dose and ADMIRE strength. In fact, this trend variation increased with both the dose and strength increase. Moreover, the SP^2^ values at the highest frequencies exceeded the level of those at the lowest frequency for high doses of 10 and 20 mGy in the soft‐tissue contrast and for every dose in the iodine contrast.

### 3.4 Slice sensitivity profile

Table 1 lists the FWHM values for the different contrast and the evaluated doses and techniques. The values for IIR are not affected by the radiation dose, although a slightly higher value resulted at 2.5 mGy, whereas ADMIRE exhibits higher FWHM of soft‐tissue contrast at low doses (ADMIRE 3: 9.2% and 6.1%, ADMIRE 5: 19.7% and 12.7% at 2.5 and 5 mGy, respectively) and lower FWHM of iodine contrast compared to that of FBP.

**Table 1:** FWHM using FBP, IIR, and AsDMIRE for different radiation doses with soft‐tissue and iodine rod.

CTDI_vol_ (mGy)	FBP	IIR	ADMIRE 3	ADMIRE 5
Soft‐tissue rod (60 HU)	FWHM^a^ (mm)			
2.5	1.221	1.267	1.333	1.461
5	1.227	1.236	1.302	1.382
10	1.227	1.220	1.262	1.302
20	1.218	1.220	1.213	1.212
Iodine rod (270 HU)	FWHM^a^ (mm)			
2.5	1.225	1.220	1.159	1.128
5	1.173	1.144	1.108	1.087
10	1.187	1.169	1.115	1.078
20	1.202	1.178	1.123	1.084

FWHM, full width at half maximum; FBP, filtered back projection; IIR, image‐based iterative reconstruction; ADMIRE, advanced modeled IR.

a Nominal thickness, 1 mm.

### 3.5 Image subtraction

**Figure 6:**
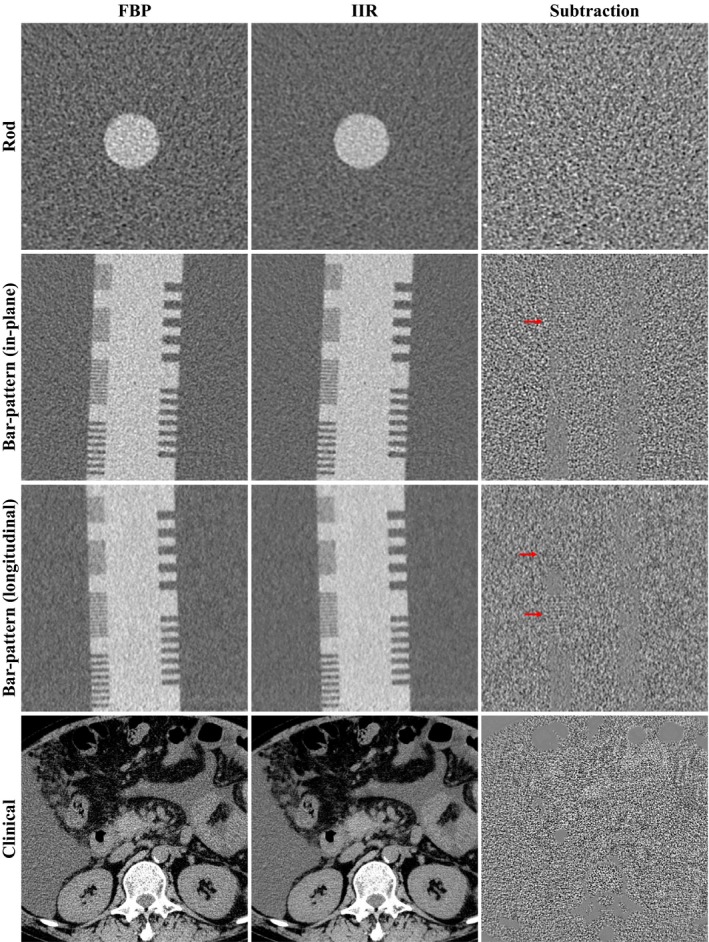
Images processed with (left column) FBP and (middle column) IIR, and corresponding subtraction images (right column), for the rod object for the TTF measurement, a bar‐pattern phantom, and a clinical abdomen image. Window width/level (WW/WL) of 200/20: FBP and IIR images. WW/WL of 40/0: subtraction images. Arrows show the fine edge structures in the subtracted bar‐pattern image

Images obtained from FBP and IIR processing, and their subtraction images are shown in Fig. 6. Only noise can be observed in the subtraction rod image from IIR, thus demonstrating its high noise removal ability to simple shape. On the other hand, the subtraction bar‐pattern phantom and clinical image show weak edge signals besides noise, thus indicating that noise removal was not completely accurate to complex patterns. The edge signals remained particularly at segments with high frequencies in the bar‐pattern phantom image. Also, fine edge patterns were detected in the clinical image. However, as these signals are weak, the IIR‐processed image does not cause notable image blurring or alteration. In addition, image regions containing bone and intestinal gas were excluded during IIR processing because they exhibit uniform areas with pixels having zero value.

## 4 DISCUSSION

We evaluated the physical image quality using IIR at four radiation dose levels on objects presenting soft‐tissue and iodine contrasts and compared the outcomes with those using FBP and ADMIRE implemented on a state‐of‐art dual source CT scanner. Overall, IIR achieved notable noise reduction and preserved both the in‐plane and longitudinal resolutions with a negligible NPS peak shift related to a change in noise texture. ADMIRE reduced image noise, but its NPS exhibited peak shifts and the resolution was strongly dependent on the dose and contrast. This trend was more remarkable when using the stronger ADMIRE 5.

Regarding noise reduction, IIR uniformly reduced the NPS over the entire spatial frequencies. No NPS peak shift occurred using IIR, except for 2.5 mGy, whereas the NPS peak of ADMIRE shifted by 20%–30% compared to FBP, confirming the values reported by Christianson et al.^9^ The peak shift causes an unnatural image texture^9^, ^14^, ^15^ and may alter the search pattern and image perception of radiologists.^14^ Therefore, IIR images might have greater clinical acceptability. However, some researchers have reported that peak shifts, like that present in the ADMIRE NPS, did not influence to diagnostic performance and instead improved low‐contrast detectability.^4^

As IIR mostly maintains the spatial resolution of FBP and reduces the NPS over the entire frequency range, its SP is uniformly better than that of FBP, thus resembling the properties achieved by dose increase. As the SP^2^ at 2.5, 5, and 10 mGy with IIR were comparable to those at 5, 10, and 20 mGy with FBP, respectively, the dose reduction with IIR was estimated at approximately 50%. However, as TTF was determined at a specific spatial resolution for a simple edge (i.e., round edge of the rod), the corresponding SP reflects the imaging performance for this limited local condition, disregarding the complicated edges of organs and tissues. Unlike IIR, SP of ADMIRE was considerably different from that of FBP. Specifically, we obtained increased SP in ADMIRE from the medium to high frequencies, with values at high frequencies exceeding those at the lowest frequency, consequently exhibiting an unrealistic trend. In fact, the rod edge is unnaturally sharp against the smoothed background, possibly related to the unusual SP trends.

The subtracted rod phantom image showing only noise suggests that IIR successfully eliminates noise. However, the subtracted bar‐pattern and clinical images showed an inhomogeneous distribution containing weak edge patterns. Hence, simple structures such as the rod might be advantageous for noise removal, whereas complete noise removal in clinical images would be more challenging. In our results, the high frequency bar‐pattern signals we tested were not completely preserved despite the pattern was simply periodic and not complicate. Although we demonstrated the dose reduction potential of IIR using physical image quality evaluation, these results may not be completely consistent with the results of clinical images. Padole et al.^6^ examined the performance of IIR and other IR techniques in submilisievert chest CT images, where IIR images were suboptimal for evaluation of subtle mediastinal structures. Thus, more conspicuous patterns in the subtraction image might appear when abdominal images acquired at low doses are processed by IIR.

## CONFLICT OF INTEREST

The authors declare no conflicts of interest associated with this manuscript.
